# Identification of COVID-19 Clinical Phenotypes by Principal Component Analysis-Based Cluster Analysis

**DOI:** 10.3389/fmed.2020.570614

**Published:** 2020-11-12

**Authors:** Wenjing Ye, Weiwei Lu, Yanping Tang, Guoxi Chen, Xiaopan Li, Chen Ji, Min Hou, Guangwang Zeng, Xing Lan, Yaling Wang, Xiaoqin Deng, Yuyang Cai, Hai Huang, Ling Yang

**Affiliations:** ^1^Department of Respiratory Medicine, Xinhua Hospital, School of Medicine, Shanghai Jiao Tong University, Shanghai, China; ^2^Department of Emergency, Xinhua Hospital, School of Medicine, Shanghai Jiao Tong University, Shanghai, China; ^3^Department of Geriatrics, Xinhua Hospital, School of Medicine, Shanghai Jiao Tong University, Shanghai, China; ^4^Department of Tuberculosis Ward 2, Wuhan Pulmonary Hospital, Wuhan, China; ^5^Center for Disease Control and Prevention, Shanghai, China; ^6^Fudan University Pudong Institute of Preventive Medicine, Shanghai, China; ^7^Warwick Clinical Trials Unit, Warwick Medical School, Coventry, United Kingdom; ^8^School of Public Health, Shanghai Jiao Tong University School of Medicine, Shanghai, China

**Keywords:** COVID-19, phenotype, treatment, principal component analysis, cluster analysis

## Abstract

**Background:** COVID-19 has been quickly spreading, making it a serious public health threat. It is important to identify phenotypes to predict the severity of disease and design an individualized treatment.

**Methods:** We collected data from 213 COVID-19 patients in Wuhan Pulmonary Hospital from January 1 to March 30, 2020. Principal component analysis (PCA) and cluster analysis were used to classify patients.

**Results:** We identified three distinct subgroups of COVID-19. Cluster 1 was the largest group (52.6%) and characterized by oldest age, lowest cellular immune function, and albumin levels. 38.5% of subjects were grouped into Cluster 2. Most of the lab results in Cluster 2 fell between those of Clusters 1 and 3. Cluster 3 was the smallest cluster (8.9%), characterized by youngest age and highest cellular immune function. The incidence of respiratory failure, acute respiratory distress syndrome (ARDS), heart failure, and usage of non-invasive mechanical ventilation in Cluster 1 was significantly higher than others (*P* < 0.05). Cluster 1 had the highest death rate of 30.4% (*P* = 0.005). Although there were significant differences in age between Clusters 2 and 3 (*P* < 0.001), we found that there was no difference in demand for medical resources.

**Conclusions:** We identified three distinct clusters of the COVID-19 patients. The results show that age alone could not be used to assess a patient's condition. Specifically, management of albumin, and immune function are important in reducing the severity of disease.

## Introduction

Since December 2019, pneumonia cases with unknown cause have been reported in Wuhan (1). It has been identified as an acute respiratory infection caused by a novel coronavirus, later named COVID-19 by the World Health Organization (2). Since that time, COVID-19 has been quickly spreading in China and other countries, making it a serious global public health threat (3). It is important for health professionals to take coordinated, timely, and effective actions to help prevent additional cases or poor health outcomes.

The entire population is generally susceptible to the virus. Confirmed cases need to be treated in designated hospitals with effective isolation and protection conditions. Critical cases should be admitted to the ICU as soon as possible (3). Mechanical ventilation, blood purification, and extracorporeal membrane oxygenation (EMCO) should be applied cautiously in severe COVID-19 patients (2). Beyond these invasive rescue methods, doctors hope to find ways to prevent disease progress from the early stage in the clinic.

Cluster analysis is one of the unsupervised learning methods which has been successfully applied in medical research (4). Cluster generation involves merging samples into larger clusters to minimize the within-cluster variations amongst patients and to maximize the between-cluster variations. Using cluster analysis, we can take advantage of in-depth phenotyping to reveal unique patterns of association among phenotypic variables (5), which may allow health professionals to develop specialized and more effective therapeutic strategies for the treatment of COVID-19 patients.

We hypothesized that COVID-19 comprises discrete clusters of patients with different clinical characteristics associated with different outcomes. To test this hypothesis, we used cluster analysis to identify COVID-19 subgroups and then determined the disease severity among subgroups. We demonstrate that this unbiased clustering approach could predict the severity of disease in patients and thus reveal the key variables clinicians could consider when evaluating patients.

## Materials and Methods

### Study Design and Participants

We conducted a retrospective, single centered and observational study in Wuhan Pulmonary Hospital, Hubei Province, China (a COVID-19-designated hospital in the epidemic outbreak) and collected clinical data from the patients diagnosed with COVID-19 between January 1 and March 30, 2020. Patients with missing clinical data were excluded.

The diagnosis and treatment of COVID-19 complied with the “new coronary pneumonia diagnosis and treatment plan” issued by the health commission of the People's Republic of China. Laboratory diagnosis of COVID-19 was confirmed by viral nucleic acid test (NAT) using high-throughput sequencing or real-time reverse-transcriptase–polymerase-chain-reaction (RT-PCR), which can amplify the open reading frame 1ab (ORF1ab) and nucleocapsid protein (NP) gene fragments of COVID-19 virus from the sputum, pharyngeal swab, or lower respiratory tract samples.

The National Health Commission of the People's Republic of China affirmed that data collection and analysis of cases and close contacts are part of ongoing investigations into outbreaks of public health events and are therefore exempt from the approval requirements of the institutional review board.

### Data Collection

Clinical data include demographic information (gender, age, comorbidities), laboratory tests (routine blood test, coagulation test, infection markers, liver and kidney function, and markers of myocardial injury), and outcomes (survival or death at hospital discharge).

### Statistical Analysis

The main factors with the highest loading in 18 variables (including all the laboratory tests) were selected using principal component analysis (PCA) at baseline. K-means cluster analysis (6), one of the most widely adopted clustering algorithms, was carried out to classify COVID-19 patients into different groups using clinical data based on the PCA results.

PCA analysis was performed using the following variables: D-Dimer, fibrinogen (FIB), activated partial thromboplastin time (APTT), prothrombin time (PT), c-reactive protein (CRP), procalcitonin (PCT), white blood cell (WBC), neutrophil count, lymphocyte count, monocyte count, alanine aminotransferase (ALT), aspartate aminotransferase (AST), albumin (Alb), helper T lymphocyte count, cytotoxic T lymphocyte count, creatinine (Cr), troponin I (TNI), and N-terminal pro-Brain Natriuretic Peptide (NT-proBNP). In order to select the number of important principal components, we chose values with an eigenvalue >1. The Oblimin method was used in the square rotation. The similarity of data was calculated using the principal factors that were identified by PCA-transformed data. Kaiser–Meyer–Olkin (KMO) and the Bartlett's test of Sphericity assessed the adaptive validity of PCA analysis. The representative variables of principal components were chosen based on their factor loading.

We performed a K-means cluster analysis in this study. The main steps were as follows: First, the initial cluster center was selected with the number of K. Second, cluster steps were repeated until cluster membership stabilized. Third, each point was assigned to its closest cluster center. Finally, the new cluster centers were computed.

SPSS version 24.0 (IBM Corp, Armonk, NY) was used for statistical analysis. Qualitative and quantitative variables were summarized using mean and standard deviation (SD), median and inter-quartile range (IQR), and number and percentage, respectively. Differences between clusters in qualitative variables were analyzed using the Chi-squared test. Differences in the quantitative variables were analyzed using the *t*-test. In the case of non-normally distributed variables, the non-parametric Mann–Whitney test was used. A *P* < 0.05 was considered statistically significant.

## Results

### Demographics and Baseline Characteristics of Patients With COVID-19

**Figure 1:**
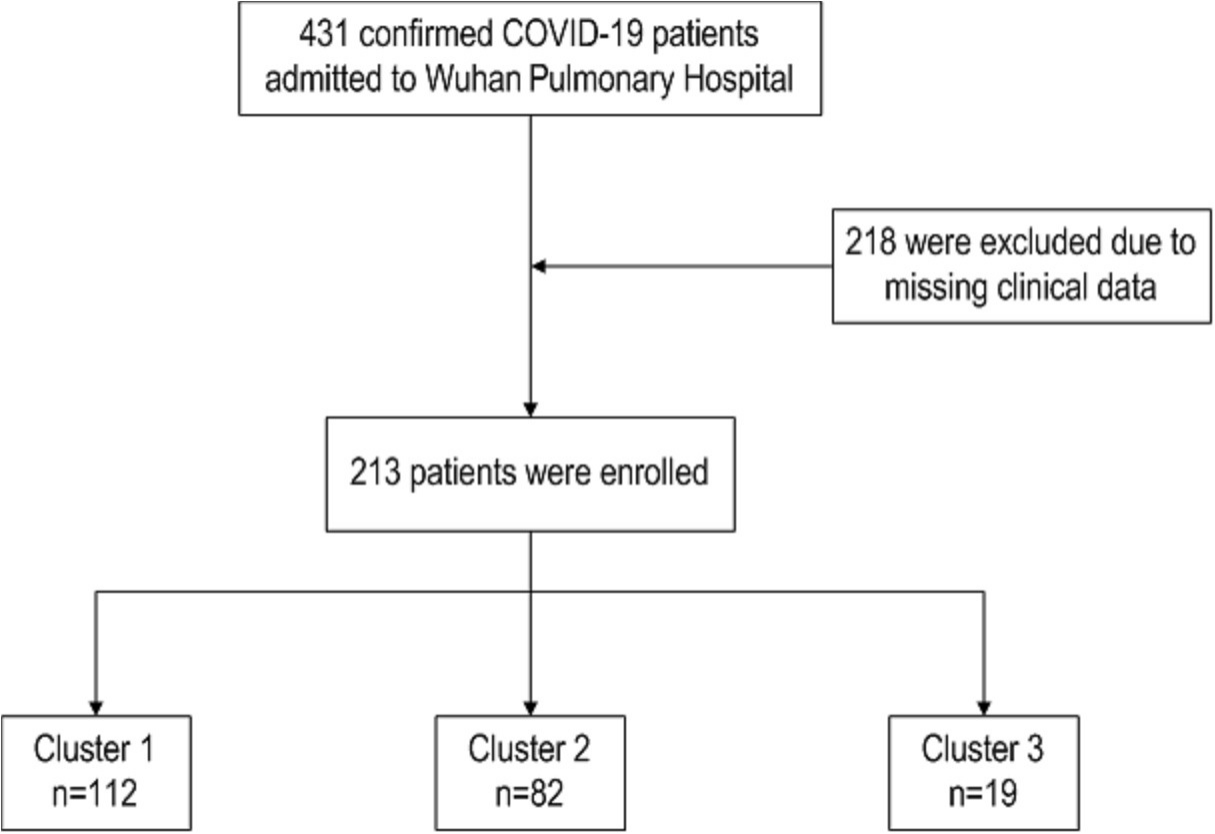
Selection of the study patients.

There were 431 confirmed COVID-19 patients admitted to Wuhan Pulmonary Hospital between January 1 and March 30, 2020 and 218 (52.8%) were excluded due to missing clinical data (Figure 1). Two hundred and thirteen patients were ultimately enrolled with a mean age of 61.85 ± 14.72 years, and 116 (54.50%) of them were males. 167 (78.40%) patients survived, while 46 (21.60%) died. Demographic characteristics, laboratory tests, and comorbidities of all patients are shown in Table 1.

**Table 1:** Baseline characteristics and laboratory tests of 213 patients.

**Characteristics**		**Count (%) or Mean (SD) or Median (IQR)**
Gender (Male, %)		116 (54.5%)
Age (years)		61.7 (14.7)
D-Dimer (mg/L)		0.5 (0.2–1.7)
FIB (g/L)		4.2 (1.4)
APTT (s)		35.8 (32.5–39.7)
PT (s)		13.1 (12.5–14.3)
WBC (×10^9^/L)		6.7 (5.1–9.2)
Neutrophil count (×10^9^/L)		5.0 (3.2–7.6)
Lymphocyte count (×10^9^/L)		0.9 (0.6–1.5)
Monocyte coun t (×10^9^/L)		0.4 (0.2)
Alanine aminotransferase (μ/L)		27 (16–41)
Aspartate aminotransferase (μ/L)		25 (17.5–42)
Albumin (g/L)		36.0 (5.4)
Creatinine (μmol/L)		68 (56–83)
Helper T lymphocyte count (n/μl)		258.3 (23.1–525.6)
Cytotoxic T lymphocyte count (n/μl)		145.4 (72.9–313.0)
CRP (mg/L)		32.4 (3.4–81.7)
PCT (ng/ml)		0.0 (0.0–0.1)
TNI (ng/ml)		0.0 (0.0–0.0)
NT-proBNP (pg/ml)		144 (34–558)
Death (*n*, %)		46 (21.6%)
Ventilator (*n*, %)	Invasive mechanical ventilation	33 (15.5%)
	Non-invasive mechanical ventilation	49 (23%)
Comorbidity (*n*, %)	Respiratory failure	37 (17.4%)
	ARDS	34 (16%)
	Heart failure	50 (23.5%)
	AKI	12 (5.6%)
	Diabetes mellitus	42 (19.7%)

*ARDS, acute respiratory dyspnea syndrome; AKI, acute kidney injury; CRP, C-reactive protein; PCT, procalcitonin; NT-proBNP, N-terminal pro brain natriuretic peptide; TNI, troponinI; FIB, fibrinogen; APTT, anginal partial thromboplastin time; PT, prothrombin time; WBC, white blood cell*.

### Principal Component Analysis and Cluster Analysis for the Identification of COVID-19 Clusters

The KMO value was 0.676, and the *p*-value of Bartlett's test of sphericity was <0.001. Six components were retained using the PCA analysis. These six components significantly contributed to explaining the relationships among the 18 variables and accounted for 73.18% of the information. The following representative variables were chosen based on relatively high factor loading: factor 1, CRP and neutrophil counts; factor 2, WBC and monocyte counts; factor 3, ALT and AST; factor 4, PCT and Fib; factor 5, TNI and D-Dimer; and factor 6, Alb and NT-proBNP (Table 2).

**Table 2:** Correlations of the 18 original variables with the six main factors derived from the principal component analysis.

	**Factor 1**	**Factor 2**	**Factor 3**	**Factor 4**	**Factor 5**	**Factor 6**
Eigenvalue	4.388	2.539	1.673	1.629	1.136	1.076
% variance explained	25.812	14.934	9.840	9.581	6.683	6.328
APTT	0.139	0.304	0.409	−0.751	−0.119	0.077
PT	0.197	0.280	0.201	−0.815	−0.113	−0.130
WBC	0.423	0.743	−0.154	0.203	−0.315	−0.043
Monocyte count	−0.254	0.646	0.033	0.242	−0.285	−0.309
Lymphocyte count	−0.696	0.514	0.261	0.028	0.058	0.072
Neutrophil count	0.603	0.592	−0.196	0.175	−0.293	−0.046
Alb	−0.707	0.068	0.146	0.018	0.017	0.174
CRP	0.747	0.014	0.232	−0.012	−0.212	0.310
ALT	0.232	−0.098	0.709	0.315	0.099	−0.369
AST	0.485	−0.047	0.705	0.218	0.295	−0.119
Cr	0.275	0.038	0.003	−0.082	0.257	−0.288
TNI	0.401	0.418	−0.084	−0.009	0.416	−0.146
PCT	0.379	0.139	0.313	0.232	0.029	0.712
Helper T lymphocyte count	−0.768	0.418	0.174	0.080	0.147	0.075
Cytotoxic T lymphocyte count	−0.761	0.425	0.182	0.105	0.039	0.065
NT-proBNP	0.473	0.368	−0.105	0.039	0.295	0.186
Fib	0.283	−0.223	0.201	0.205	−0.511	−0.130
D-Dimer	0.426	0.323	−0.282	−0.002	0.517	−0.021

*CRP, C-reactive protein; PCT, procalcitonin; NT-pro BNP, N-terminal pro brain natriuretic peptide; TNI, troponinI; FIb, fibrinogen; APTT, anginal partial thromboplastin time; PT, prothrombin time; WBC, white blood cell; Cr, creatinine; Alb, albumin; ALT, Alanine aminotransferase; AST, Aspartate aminotransferase*.

### Baseline Characteristics of COVID-19 Clusters

Three distinct subgroups were identified using the cluster analysis (Table 3). Differences between Clusters 2 and 3 are shown in Supplementary Table 1.

**Table 3:** Baseline characteristics of three clusters.

	**Cluster 1 (*n* = 112)**	**Cluster 2 (*n* = 82)**	**Cluster 3 (*n* = 19)**	***P***
Gender (Male, %)	63 (56.3%)	43 (52.4%)	10 (52.6)	0.620
Age (years)	72.7 (6.7)	54.1 (5.8)	31.4 (12.2)	<0.001
D-Dimer (mg/L)	0.9 (0.4–3.5)	0.3 (0.2–0.6)	0.3 (0.1–0.6)	<0.001
FIB (g/L)	4.1 (1.4)	4.3 (1.6)	4.1 (1.2)	0.773
APTT (s)	36.3 (23.6–40.6)	34.5 (31.8–37.2)	35.6 (33.4–41.8)	0.082
PT (s)	13.2 (12.5–14.4)	13.0 (12.4–13.9)	12.9 (12.5–13.7)	0.220
WBC (×10^9^/L)	6.7 (5.2–9.3)	6.8 (4.8–9.3)	6.3 (5.2–9.1)	0.771
Neutrophil count (×10^9^/L)	5.1 (3.7–8.0)	5.0 (3.0–7.8)	3.7 (2.8–4.9)	0.029
Lymphocyte count (×10^9^/L)	0.8 (0.5–1.3)	1.0 (0.6–1.7)	1.5 (0.9–2.3)	0.001
Monocyte count (×10^9^/L)	0.4 (0.2)	0.4 (0.2)	0.4 (0.1)	0.293
Alanine aminotransferase (μ/L)	26.5 (16.3–43.8)	28.5 (17–40.5)	19 (11–32)	0.16
Aspartate aminotransferase (μ/L)	29 (20–44.8)	23.5 (16–40.2)	19 (14–32)	0.009
Albumin (g/L)	34.8 (5.2)	37.3 (5.1)	37.9 (6.6)	0.002
Creatinine (μmol/L)	70 (58–89)	66.5 (53.8–78)	73 (52–78)	0.205
Helper T lymphocyte count (n/μl)	237.0 (85.2–422.5)	262.4 (142.7–652.7)	366.0 (274.4–696.8)	0.003
Cytotoxic T lymphocyte count (n/μl)	115.4 (51.0–239.8)	189.9 (97.8–387.8)	316.4 (164.3–498.8)	<0.001
CRP (mg/L)	44.4 (15.0–85.1)	22.6 (1.0–83.0)	19.5 (1.0–31)	0.002
PCT (ng/ml)	0.0 (0.0–0.1)	0.0 (0.0–0.1)	0.0 (0.0–0.1)	0.065
TNI (ng/ml)	0.0 (0.0–0.0)	0.0 (0.0–0.0)	0.0 (0.0–0.0)	0.015
NT-proBNP (pg/ml)	390 (94.8–875.6)	48.5 (15–188)	15 (15–292)	<0.001

*CRP, C-reactive protein; PCT, procalcitonin; NT-pro BNP, N-terminal pro brain natriuretic peptide; TNI, troponinI; FIB, fibrinogen; APTT, anginal partial thromboplastin time; PT, prothrombin time; WBC, white blood cell*.

In total, 52.6% of subjects (*n* = 112) were grouped into Cluster 1. This cluster was characterized by the oldest age with mean age of 72.7 ± 6.7 years, most obvious inflammatory reaction with the highest CRP and neutrophil count, the lowest lymphocyte count and cellular immune function and albumin level, and the highest NT-proBNP.

38.5% of subjects (*n* = 82) were grouped into Cluster 2. This cluster had the middle age with mean age of 54.1 ± 5.8 years. NT-proBNP, cytotoxic T lymphocyte count, helper T lymphocyte count, AST, and lymphocyte count fell between those of Clusters 1 and 3. CRP, Alb, and D-Dimer of Cluster 2 had a significant difference between Cluster 1. Clusters 2 was characterized by middle age and general basic situation.

Cluster 3 was the smallest cluster (*n* = 19; 8.9% of subjects). It was characterized by youngest age with mean (SD) age of 31.4 (12.2) years and highest cytotoxic T lymphocyte count.

There was no significant difference in fibrinogen, activated APTT, PT, WBC, monocyte count, ALT, creatinine, and PCT among the three clusters.

### COVID-19 Clusters and Disease Severity

The disease severity of COVID-19 in the current patient population was compared across the clusters (Table 4). Differences between Clusters 2 and 3 are shown in Supplementary Table 2. The incidence of respiratory failure, acute respiratory distress syndrome (ARDS), and heart failure in Cluster 1 was significantly higher than the other two clusters (*P* < 0.05). The proportion of non-invasive mechanical ventilation usage in Cluster 1 was 27.7%, which was significantly higher than other clusters (*P* = 0.017). Cluster 1 also had the highest death rate of 30.4% (*P* = 0.005).

**Table 4:** Disease severity of three clusters.

	**Cluster 1 (*n* = 112)**	**Cluster 2 (*n* = 82)**	**Cluster 3 (*n* = 19)**	***P***
Invasive mechanical ventilation	22 (19.6%)	10 (12.2%)	1 (5.3%)	0.056
Non-invasive mechanical ventilation	31 (27.7%)	18 (22%)	0 (0%)	0.017
Respiratory failure	30 (26.8%)	6 (7.3%)	1 (5.3%)	<0.001
ARDS	24 (21.4%)	9 (11%)	1 (5.3%)	0.019
Heart failure	36 (32.6%)	13 (15.9%)	1 (5.3%)	<0.001
AKI	9 (8%)	3 (3.7%)	0 (0%)	0.087
Death	34 (30.4%)	9 (11%)	3 (15.8%)	0.005

*ARDS, acute respiratory dyspnea syndrome; AKI, acute kidney injury*.

## Discussion

COVID-19 is a novel, rapidly spreading, viral illness that represents an emergent global health threat. Mortality rate is higher in elderly and intensive care unit (ICU) COVID-19 patients, reaching 17–38% in recent reports (7, 8). Progressive lymphocytopenia was often found in severe cases (9–11). In this study, we identified three distinct subgroups of COVID-19 through a cluster analysis of 213 patients. Cluster 1 was characterized by oldest age, highest mortality rate (30.36%), and significantly lower lymphocyte count. This result was consistent with previous reports (7, 8).

The immune system of a host controls invading pathogens and thereby determines the prognosis of patients with any infectious disease, including pneumonia (12). As immune deficiency is closely tied to mortality, evaluating the immune condition could be an important companion to monitoring a patient's general condition in order to estimate prognosis (13). We found that helper T lymphocyte count and cytotoxic T lymphocyte count in Cluster 1 were significantly lower than those of the other two clusters. This suggested more impaired immune function in the Cluster 1 patients. Treating the immune deficiency at the early stage of disease may reduce the risk of disease deterioration and improve patient prognosis. Therefore, more attention to immune function is required in the elderly, severely ill patients instead of focusing on invasive treatment only.

Low albumin can lead to hypoproteinemia, and it can cause a range of diseases, such as serous effusion, pulmonary edema, heart failure, and more. Timely correction of hypoproteinemia could effectively prevent the incidence of complications (14). Therefore we compared the albumin differences between three clusters. Albumin of Cluster 1 was significantly lower than the other two clusters in our study. Therefore, it is also important to pay attention to the albumin level in elderly patients.

Our cluster analysis suggests that immunological parameters (helper T lymphocyte count and cytotoxic T lymphocyte count) and serum albumin level are important in determining prognosis and the vulnerability to developing comorbidities, including respiratory failure, ARDS, and heart failure. Improving the immune status and albumin level of patients may be a potential measures to prevent disease progression.

The mortality rate was higher in elderly patients (7, 8). We found that the mortality rate of Cluster 3, which was characterized by the youngest mean age, was not significantly different from middle-aged patients who grouped in Cluster 2. This result aroused our attention. In previous studies, it was mentioned that some COVID-19 patients showed immune imbalance and a cytokine storm, which could be responsible for further lung injury (15–17). Young patients in Cluster 3 had the highest T lymphocyte count, and most likely had a cytokine storm. Thus, is the implication to clinicians that if a younger patient presents with COVID-19, they should check T lymphocyte counts because those with very high levels may be at risk of developing severe disease despite a younger age. This needs further pathological research to validate.

D-Dimer is a degradation product that is produced in hydrolysis of fibrin (18). Studies have reported increase in D-Dimer levels in patients with pneumonia, has an indication of the presence of thrombosis and the blood hypercoagulable state (19, 20). High D-Dimer is likely to be associated with persistent clotting disorders, microthrombotic formation, pulmonary embolism and acute myocardial infarction in long-stay patients or death patients, which may cause refractory hypoxemia, respiratory failure, disseminated intravascular coagulation or even death. Our previous study found that COVID-19 patients with higher initial and peak D-Dimer value tended to have a higher risk of death (21). In this study, we found that D-Dimer of Cluster 1 was significantly higher than other two clusters. Cluster 1 also had the highest death rate of 30.4%, which was consistent with previous studies. These patients were likely to have myocardial infarction and/or pulmonary embolism, and it might also explain the difference of myocardial enzymes (TNI and AST) among the three clusters. This might suggest the importance of early anticoagulant intervention.

Neutrophil count and lymphocyte count were found to have great prognostic power in community-acquired pneumonia. The increase of neutrophils often indicates that the patients have bacterial infection and the infection is aggravated. The decrease of lymphocyte means that the immune function is poor (22, 23). At the early stage of COVID-19, the total number of leukocytes is normal or decreases, while the lymphocyte count decreases (3). We found that Cluster 1 had the lowest lymphocyte count and the highest neutrophil count. There was no difference in Neutrophil count and lymphocyte count between Cluster 2 and 3. Our previous study found that COVID-19 patients with high neutrophil-lymphocyte Count Ratio might have a poor prognosis, even a risk of death (21). Those might suggest that the aggravated condition and the infection is difficult to control in Cluster 1.

According to our clustering results in disease severity, patients in Cluster 1 had a high incidence of respiratory failure, ARDS, heart failure, and high utilization rate of non-invasive mechanical ventilation. The demand for medical resources of these patients is significantly higher than other clusters. Thus, we suggest that Cluster 1 needs a comprehensive treatment plan, or may even need to stay in the intensive care unit. Although there were significant differences in age between Clusters 2 and 3, we also found that there was no significant difference in demand for medical resources between these two clusters. It could be interpreted that doctors should pay the same clinical attention to middle-aged and young patients. Age alone could not be used to assess a patient's condition, we must correct the misunderstanding that young patients should always be assumed to have relatively mild disease in COVID-19.

There are some potential limitations in our study. First, this was a single center retrospective study. All of the data were collected from patients in Wuhan Pulmonary Hospital. Most of the patients in this hospital were symptomatic, severe or even critical. As a result, the proportion of young and mild disease patients in the study was relatively low. Second, only 213 out of 413 patients were enrolled in our study. The exclusion of patients with missing clinical data might cause some bias in our analysis. Our results could be more representative if we are able to collect these data in the future. Finally, our data may be subjected to recall bias and selection bias due to the nature of our study. For example, the record of patients' comorbidities might not be accurate and complete, considering the unprecedented pressure during admission and treatment.

Further studies with more detailed and representative data are needed. In particular, a long-term follow up of the patients will allow us to further explore the differences between phenotypes.

## Conclusions

We identified three distinct subclasses of COVID-19 patients in Wuhan Pulmonary Hospital. It might be necessary to improve the immune function and pay attention to the underlying health conditions in the elderly patients. D-Dimer, lymphocyte count, neutrophil count, NT-proBNP, T lymphocyte count, and serum albumin should be paid attention to. This might remind us that correction of these abnormal lab results in time can be useful in preventing the corresponding complications and reducing the mortality rate. Age alone could not be used to assess a patient's condition; cluster assessment may be more reliable.

## Data Availability Statement

The original contributions presented in the study are included in the article/Supplementary Materials, further inquiries can be directed to the corresponding author/s.

## Ethics Statement

The studies involving human participants were reviewed and approved by The National Health Commission of the People's Republic of China. Written informed consent for participation was not required for this study in accordance with the national legislation and the institutional requirements. Written informed consent was not obtained from the individual(s) for the publication of any potentially identifiable images or data included in this article. Informed consent was exempted with the approval of Medical Ethics Committee of Xinhua Hospital Affiliated to Shanghai Jiaotong University School of Medicine, Shanghai, China (No. XHEC-D-2020-052).

## Author Contributions

YC, HH, and LY designed the current study and revised the manuscript. YT, GC, XLi, CJ, MH, GZ, XLa, YW, and XD collected data. WY and WL wrote the manuscript and revised the manuscript. All authors contributed to the article and approved the submitted version.

## Conflict of Interest

The authors declare that the research was conducted in the absence of any commercial or financial relationships that could be construed as a potential conflict of interest.
