# Supplementary material for: Identification of COVID-19 Clinical Phenotypes by Principal Component Analysis-Based Cluster Analysis
Source: Front Med (Lausanne). 2020 Nov 12;7:570614. doi: 10.3389/fmed.2020.570614 (PMC7690648; doi:10.3389/fmed.2020.570614)
Supplement: Supplementary file 1 [file Table_1.docx]

**STable 1. Differences of characteristics of cluster 2 and cluster 3**

|  | **Cluster2(n=82)** | **Cluster3(n=19)** | ***P*** |
| --- | --- | --- | --- |
| Age(years) | 54.1(5.8) | 31.4(12.2) | <0.001 |
| D-Dimer(mg/L) | 0.3(0.2-0.6) | 0.3(0.1-0.6) | 0.82 |
| FIB(g/L) | 4.3(1.6) | 4.1(1.2) | 0.84 |
| APTT(s) | 34.5(31.8-37.2) | 35.6(33.4-41.8) | 0.18 |
| PT(s) | 13.0(12.4-13.9) | 12.9(12.5-13.7) | 0.63 |
| WBC (*10^9/L) | 6.8(4.8-9.3) | 6.3(5.2-9.1) | 0.63 |
| Neutrophil count (*10^9/L) | 5.0(3.0-7.8) | 3.7(2.8-4.9) | 0.13 |
| Lymphocyte count (*10^9/L) | 1.0(0.6-1.7) | 1.5(0.9-2.3) | 0.11 |
| Monocyte count (*10^9/L) | 0.4(0.2) | 0.4(0.1) | 0.85 |
| Alanine aminotransferase(u/L) | 28.5(17-40.5) | 19(11-32) | 0.06 |
| Aspartate aminotransferase(u/L) | 23.5(16-40.2) | 19(14-32) | 0.18 |
| Albumin(g/L) | 37.3(5.1) | 37.9(6.6) | 0.33 |
| Creatinine(umol/L) | 66.5(53.8-78) | 73(52-78) | 0.53 |
| Helper T lymphocyte count(n/ul) | 262.4(142.7-652.7) | 366.0(274.4-696.8) | 0.13 |
| Cytotoxic T lymphocyte count(n/ul) | 189.9(97.8-387.8) | 316.4(164.3-498.8) | 0.04 |
| CRP (mg/L) | 22.6(1.0-83.0) | 19.5(1.0-31) | 0.31 |
| PCT(ng/ml) | 0.0(0.0-0.1) | 0.0(0.0-0.1) | 0.87 |
| TNI (ng/ml) | 0.0(0.0-0.0) | 0.0(0.0-0.0) | 0.09 |
| NT-proBNP(pg/ml) | 48.5(15-188) | 15(15-292) | 0.17 |

Note：CRP, C-reactive protein; PCT, procalcitonin; NT-pro BNP, N-terminal pro brain natriuretic peptide; TNI, troponinI; FIB, fibrinogen; APTT, anginal partial thromboplastin time; PT, prothrombin time; WBC, white blood cell.

**STable 2. Disease severity of cluster 2 and cluster 3**

|  | **Cluster2(n=82)** | **Cluster3(n=19)** | ***P*** |
| --- | --- | --- | --- |
| Invasive mechanical ventilation | 10(12.2%) | 1(5.3%) | 0.83 |
| Non-invasive mechanical ventilation | 18(22%) | 0(0%) | 0.02 |
| Respiratory failure | 6(7.3%) | 1(5.3%) | 0.61 |
| ARDS | 9(11%) | 1(5.3%) | 0.44 |
| Heart failure | 13(15.9%) | 1(5.3%) | 0.21 |
| AKI | 3(3.7%) | 0(0%) | 0.53 |
| Death | 9(11%) | 3(15.8%) | 0.40 |

Note：ARDS, acute respiratory dyspnea syndrome; AKI, acute kidney injury.
